# Filter Cassette Method for Analyzing Man-Made Vitreous Fibers Settled on Surfaces

**DOI:** 10.3390/ijerph16071256

**Published:** 2019-04-09

**Authors:** Tapani Tuomi, Jyrki Kilpikari, Minna Hartonen, Reima Kämppi, Heli Lallukka

**Affiliations:** Finnish Institute of Occupational Health, Topeliuksenkatu 41 B, P.O. Box 40, Työterveyslaitos, FI-00032 Helsinki, Finland; Jyrki.Kilpikari@saint-gobain.com (J.K.); minna.hartonen@ttl.fi (M.H.); reima.kamppi@ttl.fi (R.K.); heli.lallukka@ttl.fi (H.L.)

**Keywords:** exposure assessment, man-made vitreous fibers, MMVF, surface sampling

## Abstract

A new method was developed to analyze the surface count of fibers in a variety of environments. The method entails sampling surfaces with the help of suction to a filter cassette holder containing a cellulose filter. The filters were collapsed using microwave digestion in dilute acid, and the fibers filtered to polycarbonate filters, gilded, and analyzed by scanning electron microscopy (SEM). The method was compared to traditional gel tape sampling as described in International Standards Organization (ISO) standard 16000-27, following analysis with phase contrast microscopy. The methods were compared in industrial environments and in office-type environments, with the concentration range studied spanning from 0.1 to 100,000 fibers/cm^2^. The methods yielded similar results (*p* < 0.05) in concentrations from 100 to 10,000 cfu/cm^2^, while the filter cassette method gave systematically higher results in high concentrations (>10,000 cfu/cm^2^) as well as in all office-type environments studied, where the fiber count ranged from 0.1 to 20 fibers/cm^2^. Consequently, we recommend using the new method in working environments where the surface count is more than 100 fibers/cm^2^, as well as in office-type environments where the fiber count is below 10 fibers/cm^2^. It should be noted, however, that a similar limit of quantitation as with the gel tape method (0.1 fibers/cm^2^) requires sampling a minimum area of 100 × 100 cm^2^ with the fiber cassette method. Using the filter cassette method will require new guide values to be formed for office-type environments, since the results are higher than with the gel tape method. Alternatively, if present guide values or limit values are to be used with the filter cassette method, conventions as to which fiber sizes to count should be set, since SEM analysis in any case will allow for including a larger size range than phase contrast microscopy (PM). We, however, recommend against such an approach, since fibers less than 1 µm in width may not be less harmful by inhalation than larger fibers.

## 1. Introduction

Vitreous fibers produced for commercial purposes include fibrous glass and mineral wool fiber. Fibrous glass fibers and ceramic fibers have been classified by the International Agency for Research on Cancer (IARC) as “possibly carcinogenic to humans” (Class 2B), while mineral wool fibers have been grouped “unclassifiable as to carcinogenicity to humans”, causing mainly reversible irritation and inflammation of skin, eyes, and upper airways [1,2,3,4]. Mineral wool fiber is also known as man-made vitreous fibers (MMVFs). Other synonyms include synthetic vitreous fibers (SVFs), man-made mineral fibers (MMMFs), and synthetic mineral fibers (SMFs). 

Both the diameter and the length of MMVFs affect responses upon exposure of skin or airways. MMVFs are usually 2–9 µm wide with a length of 1.5–100 µm [2]. Fibers less than 3 µm in length are respirable by nose breathing, while fibers with a length of less than 5 µm can be breathed through the mouth [2]. The smaller the fiber diameter, the more likely it is to reach the lungs. A width of less than 3 µm enables the fiber to reach the alveolar region of the lungs [5]. However, MMVFs less than 5 µm are effectively dissolved or removed by alveolar macrophages. This prevents permanent damage of lung tissues that follows from, for instance, asbestos exposure [5,6]. Contrary to airway exposure, fibers more than 4 µm in length have been reported to be more harmful upon skin exposure than smaller ones [2]. Non-respirable fibers (i.e., fibers more than 5 µm in length) can collect on horizontal surfaces and may, upon redispersion or transfer by fingers to eyes, cause exposure of airways, skin, and eyes. It has been shown that the deposition velocity to the eyes increases with increasing fiber diameter. According to a convention confirmed by the World Health Organization (WHO) and the European Union Scientific Committee on Occupational Exposure Limits (EU/SCOEL), only fibers with a length above 5 µm, a width of less than 3 µm, and a length-to-width ratio of greater than 3:1 should be counted to assess occupational safety. In addition to the health consequence of exposure, this definition stems from established practices and analytical restrictions. Nevertheless, it covers the majority of MMVFs present in indoor environments or production facilities and correspond to the portion of the respirable fraction of fibrous dust able to penetrate to the alveolar region of the lungs. However, since only fibers more than 3 µm in diameter are known to cause transient irritation and inflammation of the skin, eyes, and upper airways, it is important not to exclude thick, non-respirable fibers when assessing surface contamination of fibers. In indoor air, MMVFs settled on surfaces have been reported to have diameters of 3.7 ± 2 µm and a median length of 76–86 µm [7], while, on average, longer and thicker fibers may be present in production facilities [8]. However, in practice, smaller fibers with diameters from less than 0.01 to ca. 2.5 µm can be found in mechanically ventilated offices upon electron microscopic analysis. These correspond to the fiber size distribution present in ventilation filters for incoming air and acoustic ceiling and wall panels. Such fibers are relatively harmless upon exposure of skin, eyes, or airways due to their size and elemental composition [9,10,11].

Apart from production facilities, exposure to MMVFs may be of concern in the transportation, distribution, and installation of fiber-rich products. Such commodities include acoustic ceiling and wall panels, wall insulation boards, or insulating materials of pipes and ventilation equipment. The exposure of occupants in premises where MMVF-rich materials are used is often negligible to low, but may in some instances rise to less acceptable levels. For instance, if old or broken ceiling panels or acoustic insulation materials are used, increased levels of MMVFs are present in indoor air and deposited on surfaces [7]. In addition, filters for incoming air emit fibers to indoor air, almost all of which are less than 3 µm in width and more than 50 µm in length. To the authors’ knowledge, such fibers have not specifically been reported to cause an increase in, for instance, unspecific irritation symptoms, even though mechanical ventilation may not always be unproblematic from this point of view. MMVF levels in the general air of mineral wool plants may be, according to our own measurements, 10–2000 fibers/m^3^ and, according to published data, up to 1500 fibers/m^3^ [12,13]. Construction workers working with MMVF-rich products may inhale, on average, up to 700 fibers/m^3^ during a working day and up to 1,000,000 fibers/m^3^ during tasks requiring protective masks [14,15]. The concentrations in residences and public buildings are mostly below 10 fibers/m^3^ but may in extreme cases reach concentrations up to 200 fibers/m^3^ [7,16,17].

Exposure to—or the prevalence of—fibers indoors has been estimated using diverse methods depending on prevailing concentrations as well as the presumed source of the fibers. Sampling from air, surfaces, exhaust vents, and fresh air intake vents has been used [17]. Sometimes samples are also, or exclusively, withdrawn from surfaces of ducts for incoming or outgoing air. As exposure limits or guide values have been given only for the MMVF content in air and less frequently for the surface concentration, other sampling procedures mentioned require benchmark samples of some kind. In production facilities or other occupational settings where exposure to MMVFs can be classified as acceptable to high or unacceptable, fiber concentrations are estimated from air samples using methods published by, for instance, the Health and Safety Executive (HSE), WHO, or the International Standard Organization (ISO) [18,19,20]. Samples are usually withdrawn with the help of a sampling pump on membrane filters using disposable, three-piece, conductive plastic filter holders [19]. 

In Finland, a threshold limit value of 0.2 fibers/cm^2^ has been set by the Ministry of Social Affairs and Health for the MMMF count on surfaces in indoor environments [21]. Hence, samples of dust collected for two weeks are frequently withdrawn from Finnish dwellings and offices by using sticky, gelatinous sampling tape as described by Schneider (1986). From exhaust of fresh air ducts, fiber counts in air (fibers/m^3^) have been estimated with the help of filter cloths, taking into account the duration of sampling and the laminar flow and filter dimensions [17]. Lastly, the presence or surface counts of fibers in ventilation ducts have been estimated by, for instance, wiping surfaces with sampling bags or using gelatinous tape [17].

Surface sampling with gelatinous graduated tape analyzed by phase contrast microscopy (PM) is an affordable method easy to apply in environments with relatively low surface contamination. However, it does have some limitations. The method can only be applied to smooth, clean surfaces. It has a relatively high limit of detection, stemming from the use of PM. Further, the concentration interval (fibers/cm^2^) is narrow. In practice, fiber counts of 0.1–100 fibers/cm^2^ can be analyzed. Below 0.2 fibers/cm^2^, there are less than 3 fibers per tape, rendering sampling poorly reproducible. Above 100 fibers, there are 1400 fibers per tape and 140 per band and, as a result, counting is tedious and may be unreliable. In addition, identification and counting of fibers with a length of 5 µm or less is unreliable using PM [22]. It follows that fibers between 5 and 20 µm in length are often not counted from gelatinous tapes even though they may contribute to the exposure burden and may add to the health consequence following exposure [5]. A ca. 1 cm^2^ section of the gelatinous tape may also be collapsed with a plasma asher and the residue analyzed after gilding using scanning electron microscopy (SEM) as described in ISO standard 16000-27 [23]. This enables more precise analysis covering smaller fibers, but due to the small sampling area covered, premises with low or high surface concentrations cannot be analyzed using this methodology, which all but excludes sampling from offices and production facilities.

To overcome some of the shortcomings mentioned above, the present study aimed at producing a new method for the sampling of surface contamination of MMMFs. We attempted to develop a method by which sampling is achieved using suction to a cellulose filter. After the filter is collapsed, the residue is filtered to polycarbonate membrane filters, which are gilded and analyzed with the use of SEM. The purpose was to achieve a method facilitating surface sampling on all kinds of surfaces and applicable to all working environments, with a larger sampling surface and wider concentration range, as well as a larger fiber size interval.

## 2. Methods

### 2.1. Sampling Sites

Samples were withdrawn from a plant producing acoustic ceiling and wall panels and wall insulation boards from mainly recycled glass. The portion of recycled glass was ca. 85%, constituting up to 150 million kilograms annually. The production utilized a rotary spray process, where molten glass was poured through a spinner that fiberized the glass into discontinuous fibers. Samples were taken beside the wall of a passageway adjacent to the production line, separating the manufacturing premises from a stairway leading to office areas. The sampling sites were next to the packing unit and the curing oven at a height of approx. 1½ m. In addition, samples were collected from the locker room of the male employees, from the top of the lockers at a height of approx. 2 m. The sampling sites were selected with the aim to cover the whole range of concentrations potentially present in the production facility. The concentrations were assumed to be lowest in the locker room. In the production line, concentrations were expected to decrease from the curing oven to the packing unit.

Surface samples were also taken from three mechanically ventilated office buildings, where acoustic ceiling and wall panels were used and where the sound insulation of incoming air fans may have contributed to the burden of MMVFs on horizontal surfaces. 

### 2.2. Sampling

**Figure 1:**
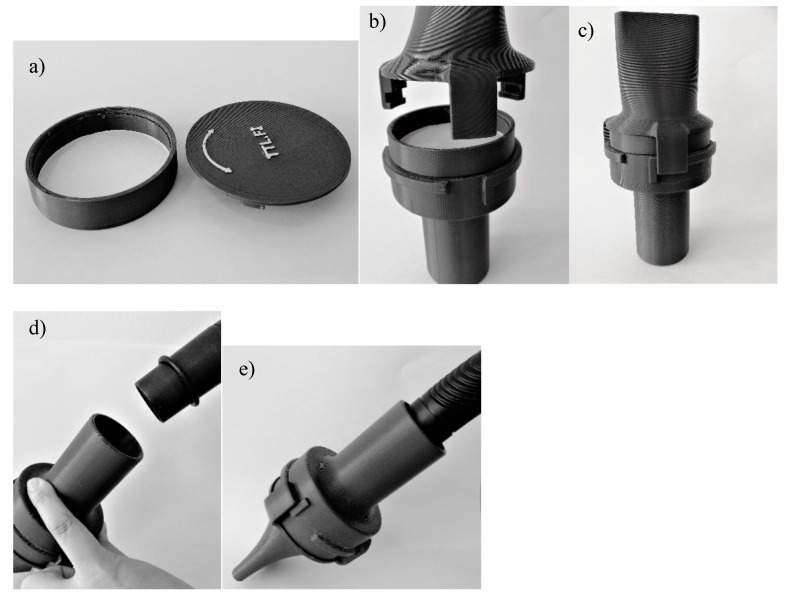
(**a**) A Whatman 3 filter paper inserted into a cassette. (**b**) The cassette inserted into a cassette holder. (**c**) A closed cassette holder. (**d**) Attaching the cassette holder to a vacuum cleaner. (**e**) Withdrawing a surface sample.

Sampling was performed by means of two methods. The first method was the one developed during the present study. The samples were taken with the help of a standard vacuum cleaner (800 w), from which the dustbag and filters for outgoing air had been removed. The vacuum cleaner was attached to a 3D-printed filter cassette holder containing a Whatman 3 (1003-070) cellulose filter, 70 mm in diameter with a pore size of 6 µm (Merck KGaA, Darmstadt, Germany). See Figure 1a–e for a detailed description of the filter cassette holder with accompanying parts and its attachment to the vacuum cleaner. Samples were taken from smooth surfaces cleaned by wet wiping 14 days prior to sampling. Rectangular templates with a minimum size of 20 × 20 and maximum size of 120 × 120 cm were used to ensure that predefined sampling areas were met. The templates had a collar with a minimum width of 5 cm to inhibit drawing of fibers from outside the sampling range. In industrial premises, four parallel samples were taken from each sampling site, while only one sample was withdrawn from each sampling location in most of the offices studied.

For reference, sampling was performed also as described in ISO standard 16000-27 [22] using adhesive tapes with a surface area of 14 cm^2^ (BM-Dustlifters, BM Environmental Engineering, BVDA International BV, Haarlem, The Netherlands). Four parallel samples were taken from each sampling sites, both in office-type environments and the production plant.

### 2.3. Preparation of Filter Samples

Filter cassettes were opened and the filters transferred to clean polytetrafluoroethylene (PTFE) digestion vessels. The cassette internal surfaces were rinsed with 5 ml of deionized water and the water collected in the digestion vessels before adding (slowly) 5 mL of concentrated nitric acid. The vessels were capped using a torque wrench and the samples digested: 1200 W power, temperature gradient from 22 °C to 215 °C during 12 min, the temperature held for 10 min at 215 °C, and the samples cooled for at least 5 min before transferring to a fume hood. Samples were opened at the earliest 30 min before continuing. The samples were rinsed from the PTFE tubes using deionized water and the volume adjusted to 200 ml before filtering the samples through a polycarbonate filter (0.8 µm pore size, 37 mm diameter, Isopore track-etched membrane filters or 0.8 µm pore size, 25 mm diameter, Nuclepore track-etched membrane filters, Merck KGaA, Darmstadt, Germany) using suction. When necessary, a 1:10 and/or 1:100 dilution was prepared using deionized water prior to filtering. Filtered samples were gilded prior to analysis using a Bal-Tec SCD 050 device (BalTec Maschinenbau AG, Pfäffikon, Switzerland).

### 2.4. Analysis of Filter Samples

The gilded polycarbonate filters were analyzed as described in ISO standard 14966 [19]. Briefly, ca. one-quarter portions of the filters were cut out and the pieces analyzed using a Quanta 200 FEG (Thermo Fischer Scientific, Massachusetts, USA) or a JSM 6610 LA (JEOL Technics Ltd, Tokyo, Japan) scanning electron microscope. A magnification of 500 was used to count the fibers and 50–100 image fields were counted. The size of the image field used to calculate fiber counts per square µm and, hence, per sample was adjusted according to the magnification used. Fibers were identified based on morphology and dimensions, using the WHO criteria (L > 5 µm, W < 3 µm, W:L > 3:1). All MMVF fibers meeting the WHO criteria mentioned above, but including fibers more than 3 µm in width and excluding fibers <0.5 µm in width, were counted. In uncertain cases, MMMF fibers were identified by obtaining energy dispersive x-ray (EDX) spectra and comparing the Si:Ca:Al:Na/Mg ratio to reference spectra.

### 2.5. Analysis of Adhesive Tapes

The tapes were counted under a stereomicroscope (Leica MZ12, Wetzlar, Germany) using a magnification of 100. A graticule slide with twenty lines (ten bands) per 20 mm was used underneath the tape and the magnification adjusted to cover a diameter corresponding to ca. 2 mm (the space between bands) to facilitate counting. All MMVF fibers meeting the WHO criteria mentioned above, but including fibers more than 3 µm in width, were counted. 

### 2.6. Testing the Difference between Method-Specific Data and the Correlation between the Methods

Standard t-tests with a 95% confidence interval were used to compare results derived with the two methods in different concentration ranges. 

A linear regression analysis was performed with data points derived with the two methods. The Pearson correlation coefficient was tested against its sample-size-dependent *p*-value (critical value, 95% level of significance, two-tailed test) as described by Warner [24]:

(1) $$\rho_{x,y}\;=\;\sqrt{t^{2}/\left(t^{2}+\;n\;-\;2\right)}$$

at the *t*-test critical value (*t*) corresponding to the relevant degree of freedom.

### 2.7. Assessing the Fiber Size Range in a Novel Acoustic Panel and a Filter for Incoming Air

Samples were cut out from an Ecophon Gedina AT24 PE acoustic panel (Saint-Gobain Ecophon Oy, Hyvinkää, Finland) as well as a Camfil F7 ventilation filter for incoming air (Camfil Svenska AB, Trosa, Sweden). Samples were dissolved in deionized water with the help of an Instrusonic model W181F ultrasound bath (Ultrasonic Finland Ltd, Lahti, Finland) and filtered onto 37 mm membrane filters, gilded, and analyzed, as described above.

## 3. Results and Discussion 

### 3.1. Method Equivalence and Performance in Industrial Settings

On average, concentrations in all sampling sites examined were lower when using the adhesive tape method than with the new filter cassette method developed (Table 1). One explanation is that during the 14-day sampling time, there were enough dust and fibers deposited on surfaces to overload the tapes and, hence, we were unable to pick up all dust on surfaces with one tape. This can in lower concentrations be circumvented by repeating the sampling with new gel tapes on the same surface, until the surface is clear from all dust [23]. In addition, as the tapes contained from a minimum of 765 (locker room) to 496,000 (curing) fibers, it is evident that the fibers were often layered and we were unable to count all of them. Even so, for up to a concentration of 20,000 fibers/cm^2^ the two methods yielded results that were not significantly different upon statistical comparison (t-test, *p* < 0.05, see Table 1). Consequently, the deviation of results from one method to the other is not significantly higher than the method specific standard deviations in concentrations from 80 to 20,000 fibers/cm^2^, but clearly increases with increasing concentrations (Table 1). However, in practice, adhesive tapes are very tedious to analyze when the fiber concentration exceeds 100 fibers/cm^2^ and virtually impossible when the concentration approaches 10,000 fibers/cm^2^ and as can be seen from the deviation of the results between methods, when the concentration approaches 50,000 fibers per cm^2^, the capacity of the tapes is surpassed (Table 1). 

### 3.2. Method Equivalence and Performance in Office-Type Environments

**Figure 2:**
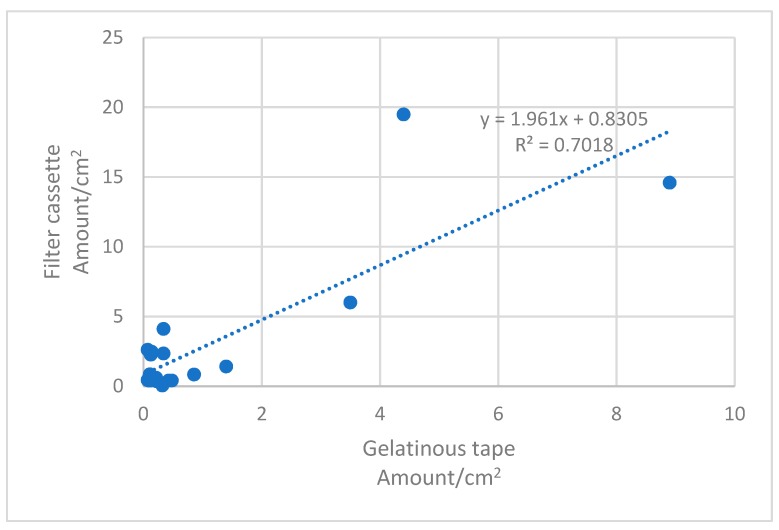
Fiber counts in office-type environments using the adhesive tape method (x) and the filter cassette method (y) (*n* = 20). The dotted line represents the regression line (y = 1.96x + 0.83).

**Figure 3:**
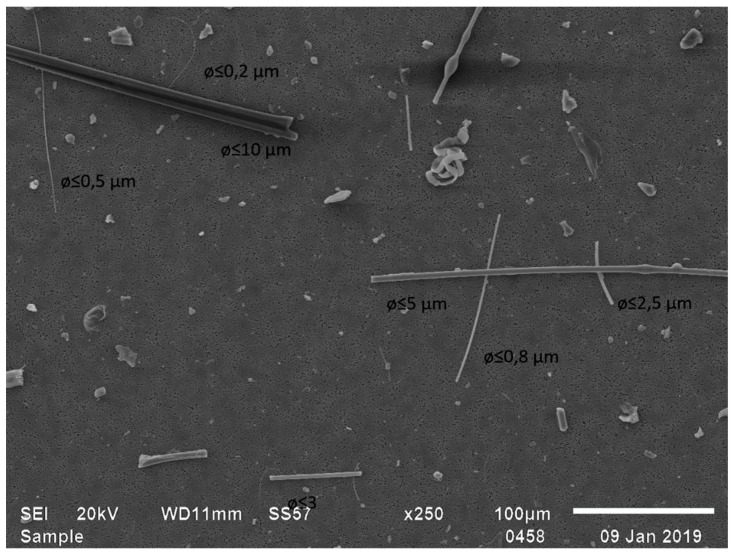
SEM image of fibers derived from Ecophon Gedina AT24 PE acoustic panels, Saint-Gobain Ecophon Oy, Finland.

**Figure 4:**
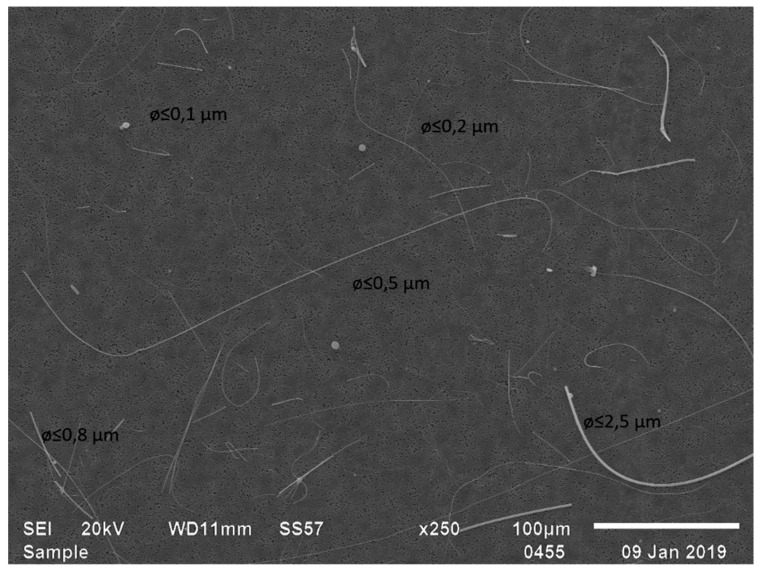
SEM image of fibers derived from Camfil F7 ventilation filters for incoming air, Camfil Svenska AB, Sweden.

As in the production plant samples, concentrations in office-type environments were systematically higher with the filter cassette method than with the adhesive tape method (Table 1, Figure 2). The most likely explanation is that fibers from 0.5 to 1 µm in width, present in all filter cassette samples analyzed by SEM (Figure 3 and Figure 4), may remain undetected by PM from the gel tapes. Similar results have previously been presented by Vallarino et al. [25], who were unable to detect fibers smaller than 0.85 µm in diameter from gel tapes by PM. There was a statistically significant correlation between the methods (*p* < 0.05; R^2^ = 0.70), with the filter cassette method (y) yielding results, on average, 63% higher than the gel tape method (x) in a concentration interval from 0.05 to 20 fibers/cm^2^ (y = 1.96 + 0.83 fibers/cm^2^; see Figure 2).

In a narrow concentration interval typical for modern mechanically ventilated offices in Finland (0.1–0.2 fibers/cm^2^, using the gel tape method), the within-method deviation was higher when using the gel tape method than when using the filter cassette method (Table 1). In addition, the within-method deviation of gel tape results was even higher than the deviation of results between the methods. This is likely explained by the small area sampled by the gelatinous tape, yielding a high deviation in low concentrations due to random deposition of fibers. Due to this, the ISO recommends a minimum of three parallel samples in small offices with a surface area of less than 30 m^2^ and a minimum ten parallel samples in large spaces [23].

### 3.3. The Limits of Detection and Quantitation of the Filter Cassette Method

When counting 100 SEM image fields per sample with a magnification of 500, the area covered is ca. 0.02 cm^2^ of an analytical polycarbonate filter with a diameter of 3.7 cm (effective diameter of 3.1 cm) and an effective area of 7.55 cm^2^. This means that to reach a similar limit of detection (LOD) as with the adhesive tape method, when covering a sampling area of 14 cm^2^ when counting ca. 100 microscopic fields per tape, an area of 40 × 40 cm^2^ needs to be sampled on the filters (Table 2). This will yield an LOD of 0.1 fibers/cm^2^. To reach a lower LOD, a smaller analytical filter and/or a larger sampling area can be used (Table 2). Using an analytical filter with a diameter of 2.5 cm (effective diameter of 2.1 cm) will yield an LOD of 0.03 fibers/cm^2^ with a sampling area of 50 × 50 cm^2^, when counting 100 microscopic fields (Table 2). Correspondingly, with the same-sized analytical filter, a sampling area of 100 × 100 cm^2^ will yield an LOD of 0.01 fibers/cm^2^ (Table 2). This corresponds to 1/10 of the LOD of the adhesive tape method.

When assessing the limit of quantitation (LOQ) of the filter cassette method, the fiber count of blanks extracted and analyzed identically to native samples needs to be accounted for. MMVFs are ubiquitous in most laboratory environments. In laboratories, MMVFs may be emitted from the ventilation system in higher concentrations than in offices, because the ventilation rate is higher. In addition, fibers are carried to laboratory premises on workers’ clothes and other textiles. HEPA air filters as well as acoustic and thermal insulation used to control equipment-derived noise and heat may also act as sources of MMVFs in laboratory environments. Hence, blanks may be contaminated with fibers originating from sampling filters used (i.e., from production and packing of filters), from filter cassettes and PTFE digestion vessels used (i.e., from laboratory equipment maintenance). In addition, sample-to-blanks or air-to-blanks contamination of fibers may occur during the filtering of digested samples as well as while preparing the samples for gilding. When taking into account the LOD, as well as the standard deviation of blanks, with a confidence limit of 95% (LOD + t_0.05_*std), a LOQ similar to the LOQ (=LOD) of the gel tape method, 0.1 fibers/cm^2^ requires a sampling area of 100 × 100 cm^2^ with the filter cassette method (Table 2).

In other words, due to the presence of background fibers on every filter analyzed, the LOQ of the filter cassette method was not better than the LOQ of the gel tape method. To reach an LOQ similar to the gel tape method, an area of 100 × 100 cm^2^ needs to be sampled with the filter cassette method, provided an analytical filter with a diameter of 2.5 cm is used. If a larger analytical filter is used or a smaller sampling area is covered, the LOQ of the filter cassette method will decrease (Table 2). One implication is that when using the filter cassette method in office-type environments, a minimum sampling area of 100 × 100 cm^2^ is recommended.

### 3.4. Other Practical Considerations

In the present study, a magnification of ca. 250 was used when analyzing the adhesive tapes using optical microscopy. Fibers with a size of ≥20 µm in length and ≤3 µm in width, with a length-to-width ratio of ≥3:1, are routinely counted to assess mineral fiber concentrations in Finland by using optical microscopy [21]. In the present study, we included fibers with a length of 5–20 as well, the width-to-length ratio of which was often difficult to assess with an optical microscope. When using SEM, we used the same criteria for the length and length-to-width ratio. However, fibers less than 0.5 µm in width were excluded, which accounted for most of the fibers originating from the filters for incoming air (Figure 4). This was done because such fibers are not visible with the optical microscope even though in many office-type environments analyzed during the present study, fibers less than 0.5 µm in width constituted a majority of the fibers visible in the filter samples analyzed by SEM. Previously, during ceiling installation using mineral wool, ca. 17 % of fibers analyzed by SEM, were reported to be <0.5 µm in diameter [26]. 

To avoid contamination, before and after sampling, each filter cassette holder should be contained in individual clean plastic bags suited for analytical purposes, such as zipper-top polyethylene bags. Contamination of used materials particularly with small fibers stemming from ventilation systems for incoming air (see Figure 4) is a major concern. Hence, prior to use, filters, cassettes, and cassette holders should be stored in, for instance, clean, contained boxes or plastic bags. For each sampling site and/or every five samples, blank (field) samples should be prepared. Blanks are stored and treated identically to other samples and their results should be subtracted from the results of native samples.

Regardless of the method used, when sampling fibers from surfaces in office-type environments, it should be considered that fibers are usually unevenly distributed. The reasons are manifold. The concentration is dependent on the closeness to the source. For instance, concentrations are usually higher near the vents for incoming air if a large portion of the fibers originate from the ventilation systems. Fibers will usually deposit close to the source of origin, such as a broken acoustic panel. However, the flow of air will to some extent affect the deposition of fibers on surfaces, and in some cases, the resuspension of fibers to air. Consequently, two surface samples will never be truly parallel when the surface concentration is low, and when the samples are small in area and/or far apart. Due to these considerations, ISO standard 16000-27 requires attaining a minimum of three parallel gel tape samples from small offices and, in offices with a surface area of more than 30 m^2^, a minimum of five parallel samples [23]. It follows that sampling a larger area as is the case with the filter cassette method—a minimum of 10,000 cm^2^ as opposed to 14 cm^2^ with gel tapes—will lower the within-method deviation and decrease the need for parallel sampling (Table 1).

## 4. Conclusions

In environments where the MMVFs present are predominantly more than 1 µm in diameter, and where the fiber count on surfaces is 80–10,000 fibers/cm^2^, the two methods compared yielded similar results. This was the case, however, in mechanically ventilated office-type environments. In these, the surface concentration of fibers is usually below 10 fibers/cm^2^, and the within-method standard deviation of the gel tape method results high, due to the small area sampled in combination with the uneven distribution of fibers on surfaces. In addition, fibers less than 1 µm in diameter, possibly originating from ventilation filters, may constitute a major portion of fibers present. Even though we excluded fibers less than 0.5 µm in width in the SEM analyses, the results in office-type environments were systematically higher with the filter cassette method when compared to the gel tape samples analyzed by PM. This may be explained by the fact that fibers from 0.5 to 1 µm in width may remain undetected by PM from the gel tapes. When also considering that the within-method deviation of the gel tape method in low concentrations was high, necessitating a minimum of three parallel samples, we recommend using the filter cassette method in office-type environments where the fiber count is below 10 fibers/cm^2^.

In addition, gel tapes become overloaded in high surface concentrations and, therefore, the filter cassette method is the method of choice in industrial environments and in other environments where the surface count of fibers is more than 100 fibers/cm^2^. When a limit of quantitation of 0.1 fibers/cm^2^ needs to be achieved, a sampling area of 100 × 100 cm^2^ and an analytical filter with a maximum width of 2.5 cm should be used with the filter cassette method.

Two surface samples will never be truly parallel when the surface concentration is low and when the samples are small in area and/or far apart. Using the filter cassette method will, however, help to overcome some, but not all, of these sources of error because the sampling surface is much larger than with the gel tape method.

Sampling and preparation of samples with the filter cassette method is clearly more laborious than with the gel tape method. However, as the gel tape method requires a minimum of three parallel samples due to the deviation of results in parallel samples, and since both methods require counting an identical number of microscopical fields, counting of fibers with the filter cassette method will take up ca. one-third of the time required to analyze gel tapes. It should be considered, however, that the filter cassette method requires analyzing a minimum of one field blank per sampling site and/or one blank for each five samples.

Using the filter cassette method will require new guide values to be formed for office-type environments, since the results are higher than with the gel tape method. Alternatively, if the present values are to be used with the filter cassette method, conventions as to which fiber sizes to count should be set, since SEM analysis in any case will allow for including a larger size range than PM. We would, however, recommend against such an approach, since fibers less than 1 µm in width may not be less harmful by inhalation than larger fibers.

## Figures and Tables

**Table 1:** Deviation of results between methods vs method-specific relative standard deviations in samples from production plant and open space office.

Sampling Site ^1^	Mean Concentration, Gel Tape (Fibers/cm^2^)	Mean Concentration, Filter Cassette (Fibers/cm^2^)	Relative Difference in Results ^2^ (%)	% RSD, Gel Tape	% RSD, Filter	Concentration Range ^3^ (Fibers/cm^2^)
Dressing room	101	108	−7	19	24	87–137
Packing	13,400	16,500	−21	29	27	12,000–36,300
Curing	28,200	107,500	−117	32	26	70,600–13,800
Open-plan office	0.17	0.47	−47	49	26	0.36–0.60

^1^ Number of samples per sampling site were 4 per method; ^2^ [average (tape) + average (filter)]/average (tape + filter); ^3^ from filter cassette method results.

**Table 2:** Limits of detection (LODs) and quantitation (LOQ) of the filter cassette method.

Analytical Filter Diameter (cm)	Area Sampled (cm^2^)	Fields Counted	Theoretical LOD (Fibers/cm^2^)	LOQ Measured from Blanks ^3^
3.7 ^1^	20 × 20	50	0.82	9.00
3.7	40 × 40	50	0.20	2.26
3.7	50 × 50	50	0.13	1.44
3.7	100 × 100	50	0.03	0.36
3.7	20 × 20	100	0.41	9.00
3.7	40 × 40	100	0.10	2.26
3.7	50 × 50	100	0.07	1.44
3.7	100 × 100	100	0.02	0.36
2.5 ^2^	20 × 20	50	0.38	3.90
2.5	40 × 40	50	0.09	0.96
2.5	50 × 50	50	0.06	0.62
2.5	100 × 100	50	0.02	0.15
2.5	20 × 20	100	0.19	3.90
2.5	40 × 40	100	0.05	0.96
2.5	50 × 50	100	0.03	0.62
2.5	100 × 100	100	0.01	0.15

^1^ Effective diameter = 3.1 cm; ^2^ effective diameter = 2.1 cm; ^3^ nine blanks from three different analysis sequences.
